# Association of Persistent and Severe Postnatal Depression With Child Outcomes

**DOI:** 10.1001/jamapsychiatry.2017.4363

**Published:** 2018-01-31

**Authors:** Elena Netsi, Rebecca M. Pearson, Lynne Murray, Peter Cooper, Michelle G. Craske, Alan Stein

**Affiliations:** 1 Department of Psychiatry, University of Oxford, Oxford, United Kingdom; 2 School of Social and Community Medicine, University of Bristol, Oakfield House, Bristol, United Kingdom; 3 School of Psychology and Clinical Language Sciences, University of Reading, Reading, United Kingdom; 4 Department of Psychology, Stellenbosch University, Matieland, Stellenbosch, South Africa; 5 Department of Psychology, University of Cape Town, Rondebosch, Cape Town, South Africa; 6 Department of Psychology, University of California, Los Angeles; 7 Department of Psychiatry and Biobehavioral Sciences, University of California, Los Angeles; 8 Medical Research Council/Wits Rural Public Health and Health Transitions Research Unit (Agincourt), School of Public Health, Faculty of Health Sciences, University of the Witwatersrand, Johannesburg, South Africa

## Abstract

**Importance:**

Maternal postnatal depression (PND) is common and associated with adverse child outcomes. These effects are not inevitable, and it is critical to identify those most at risk. Previous work suggests that the risks of adverse outcomes are increased when PND is severe and persistent, but this has not been systematically studied.

**Objective:**

To examine the association between differing levels of persistence and severity of PND and long-term child outcomes.

**Design, Setting, and Participants:**

The sample for this observational study comprised participants in the Avon Longitudinal Study of Parents and Children in the United Kingdom. Three thresholds of PND severity—moderate, marked, and severe—were defined using the self-rated Edinburgh Postnatal Depression Scale (EPDS). Depression was defined as persistent when the EPDS score was above the threshold level at both 2 and 8 months after childbirth. For each of these severity and persistence categories, the following were examined: (1) the trajectories of later EPDS scores (6 time points between 21 months and 11 years after childbirth) and (2) child outcomes—behavioral problems at 3.5 years of age, school-leaving mathematics grades at 16 years of age, and depression at 18 years of age. Data analysis was conducted from July 12, 2016, to February 8, 2017.

**Main Outcomes and Measures:**

Child behavioral problems at 3.5 years of age using the Rutter total problems scale, school-leaving mathematics grades at 16 years of age extracted from records of external national public examinations, and offspring depression at 18 years of age using the Clinical Interview Schedule–Revised.

**Results:**

For the 9848 mothers in the sample, the mean (SD) age at delivery was 28.5 (4.7) years. Of the 8287 children, 4227 (51%) were boys and 4060 (49%) were girls. Compared with women with PND that was not persistent and women who did not score above the EPDS threshold, for all 3 severity levels, women with persistent PND showed elevated depressive symptoms up to 11 years after childbirth. Whether persistent or not, PND doubled the risk of child behavior disturbance. The odds ratio (OR) for child behavioral disturbance for mothers with moderate PND was 2.22 (95% CI, 1.74-2.83), for mothers with marked PND was 1.91 (95% CI, 1.36-2.68), and for mothers with severe PND was 2.39 (95% CI, 1.78-3.22). Persistence of severe PND was particularly important to child development, substantially increasing the risk for behavioral problems at 3.5 years of age (OR, 4.84; 95% CI, 2.94-7.98), lower mathematics grades at 16 years of age (OR, 2.65; 95% CI, 1.26-5.57), and higher prevalence of depression at 18 years of age (OR, 7.44; 95% CI, 2.89-19.11).

**Conclusions and Relevance:**

Persistent and severe PND substantially raises the risk for adverse outcome on all child measures. Meeting criteria for depression both early and late in the postnatal year, especially when the mood disturbance is severe, should alert health care professionals to a depression that is likely to be persistent and to be associated with an especially elevated risk of multiple adverse child outcomes. Treatment for this group should be prioritized.

## Key Points

**Question:**

What is the association of differing levels of persistence and severity of postnatal depression with long-term child outcomes?

**Findings:**

This observational study of 9848 women with varying levels of postnatal depression and 8287 children found that, compared with children of women with postnatal depression that did not persist, of either moderate or severe intensity, children of women with persistent and severe depression are at an increased risk for behavioral problems by age 3.5 years as well as lower mathematics grades and depression during adolescence. Furthermore, women with persistent postnatal depression are likely to experience significant depressive symptoms until at least 11 years after childbirth.

**Meaning:**

Women with persistent and severe postnatal depression should be prioritized for treatment because they are likely to continue to experience high levels of depressive symptoms and because of the high risk of adverse child development.

## Teaser

This study uses data from a longitudinal study to examine the association between chronic and severe postpartum depression in mothers and adverse outcomes in their children.

## Introduction

Postnatal depression (PND), also known as postpartum depression, is common, affecting approximately 10% of women in high-income countries,^1^ with higher estimates in low- and middle-income countries.^2^ Extensive research documents the association of PND with child development, including delayed cognitive and language development, higher rates of behavioral problems, insecure or disorganized attachment, lower school-leaving grades (ie, General Certificate of Secondary Education [GCSE] examinations in the United Kingdom) at 16 years, and higher rates of depression at 16 to 18 years of age.^3,4,5,6^ An economic analysis suggests that, in the United Kingdom alone, the long-term costs of perinatal maternal mental health disorders may reach up to £8.1 billion (US $10.8 billion as of December 2017) per year for every 1-year birth cohort, principally because of the impact on the children.^7^

Two factors suggested to be important in determining child outcomes in the context of maternal depression are the chronicity and severity of the maternal mood disorder, but data are limited for PND specifically.^8,9,10,11,12,13,14^ Campbell and colleagues^15^ found an increased risk for the child if PND persisted to 6 months after childbirth, when infants of mothers with persistent depression were less positive during interactions than infants of women with either no depression or remitted depression. Similarly, Petterson and Albers^8^ reported that, in a sample of children younger than 5 years, compared with nonexposed children, those exposed to persistent maternal depression had lower cognitive scores (a decrement of 0.47 SDs for girls and 0.36 for boys). Beyond the perinatal period, the Sequenced Treatment Alternatives to Relieve Depression (STAR*D) Child studies, which examined depression in mothers of school-aged children, have shown that the risk of offspring depression increased markedly with both the chronicity and severity of maternal depression.^16^ In summary, evidence suggests that both persistent PND and severe PND raise the risk of adverse child outcomes.^3,8,9,10^

However, PND that is both severe and persistent has not been well described in the literature, as highlighted by a recent report for the US Preventive Services Task Force.^17^ Studies have generally included small sample sizes, had short-term follow-up periods, and examined a limited range of outcomes. Systematic research into this question is therefore required to establish the precise duration and symptom thresholds of significance for compromised child functioning. Elucidating the relative magnitude of the consequences for children of different levels of PND severity and persistence could help with targeting intervention resources, thereby limiting the time children are exposed to maternal depression and potentially compromised parenting.

Using participants in the Avon Longitudinal Study of Parents and Children (ALSPAC), we investigated the sequelae of PND on subsequent maternal depression and child outcome. The ALSPAC involves multiple assessments of maternal depression over the first 18 years of offspring life, including 2 postnatal assessments. Our study had 2 aims: (1) to examine the natural course of different levels of PND severity identified at 2 and 8 months after childbirth using growth curve modeling and (2) to examine the association of PND persistence and severity with child behavioral problems at 3.5 years of age, GCSE mathematics grades at 16 years of age, and self-reported depression at 18 years of age. These 3 offspring outcomes have been shown to be associated with maternal PND in this sample.^18,19,20^ We hypothesized that (1) women with persistent and severe PND would continue to show elevated levels of depression at the following assessments and (2) children of women with persistent and severe PND would be at an increased risk for behavioral problems, lower mathematics grades, and higher rates of depression.

## Methods

### Sample

Our study sample comprised participants in the ALSPAC cohort (see the eAppendix in the Supplement for full details).^21^ Ethical approval for the study was obtained from the ALSPAC Law and Ethics Committee and the local research ethics committees. All participants provided written informed consent. Data analysis was conducted from July 12, 2016, to February 8, 2017.

We had complete data on maternal depression in the postnatal year for 9848 mothers and at 11 years after childbirth for 6182 mothers. Data were available on child behavioral problems at 3.5 years of age for 8419 children, on GCSE mathematics grades at 16 years of age for 5198 children, and on offspring depression at 18 years of age for 3613 children.

### Depression Measures

Maternal depression was measured using the self-rated Edinburgh Postnatal Depression Scale (EPDS; score range: 0-30, with higher scores indicating more severe depressive symptoms).^19,22^ We defined 3 levels of PND severity: 13 to 14 points indicating moderate depression; 15 to 16 points, marked depression; and 17 or more points, severe depression. These threshold levels are consistent with those in previous analyses^23^ and have been shown to have high specificity and positive predictive value for major depressive disorder (for ≥13 points: 95.7% and 66.7%; for ≥15: 99.3% and 82.7%; and for ≥17 points: 99.7% and 92.4%, respectively).^24^ The thresholds identified 3 groups of approximately equal size at both 2 and 8 months (ie, at 8 months, 8.7% of the population scored ≥13 points; of those, 3.3% scored 13-14 points, 2.2% scored 15-16 points, and 3.2% scored ≥17 points). Depression was assessed at 2, 8, 21, 33, 61, 73, 93, and 134 months after childbirth to establish the natural course of PND.

Postnatal depression was identified as persistent when an individual scored above the EPDS threshold at both the 2- and 8-month postnatal assessment. Postnatal depression was considered not persistent when someone scored above the threshold only at the 2-month postnatal assessment. In this way, we were able to examine the association of chronicity with the outcomes at different levels of PND severity.

We examined the association of PND with the outcomes for the following 7 mutually exclusive groups: (1) no depression—reference group (EPDS score of <13 points in the postnatal year), (2) moderate but not persistent depression (EPDS score of 13-14 points at 2 months; EPDS score of <13 points at 8 months), (3) marked but not persistent depression (EPDS score of 15-16 points at 2 months; EPDS score of <15 points at 8 months), (4) severe but not persistent depression (EPDS score of ≥17 points at 2 months; EPDS score of <17 points at 8 months), (5) moderate and persistent depression (EPDS score of 13-14 points at 2 months; EPDS score of ≥13 points at 8 months), (6) marked and persistent depression (EPDS score of 15-16 points at 2 months; EPDS score of ≥15 points at 8 months), and (7) severe and persistent depression (EPDS score of ≥17 points at 2 and 8 months).

### Maternal Measures

Mothers participating in the ALSPAC indicated their highest education level on a questionnaire, which was sent out in their last trimester of pregnancy (eAppendix in the Supplement). Mothers with education continuing beyond 16 years of age were categorized as having high education (3841 [39%] of the sample), and those with education up to 16 years of age were categorized as having low education (6007 [61%]).

### Child Measures

#### Rutter Total Problems Scale

Child behavioral problems at 3.5 years of age were assessed by maternal report using the total problems scale (sum of hyperactivity and emotional and conduct problems) of the Rutter revised preschool questionnaire.^25^ The measure was split into quartiles.

#### School-Leaving Mathematics Grades

Grades achieved in mathematics were extracted from records of the GCSE, which are external national public examinations taken at 16 years of age at the end of high school in the United Kingdom. The accepted binary coding was the achievement of an A* to a C grade (coded as 0) or no such achievement (coded as 1).

#### Offspring Depression

Offspring depression was assessed at 18 years of age using the Clinical Interview Schedule–Revised, a self-administered computerized interview.^26^ A binary variable indicating a diagnosis of depression on the Clinical Interview Schedule–Revised or no such diagnosis was the outcome measure (eAppendix in the Supplement).

### Confounding Variables

Owing to the relatively small sample size of mothers with PND that was persistent and marked (n = 75) or severe (n = 83), it was important to maintain the sample size and minimize the complexity of statistical models. Therefore, in the adjusted models, we controlled only for maternal education, the only variable in this sample shown to considerably influence the association between PND and child outcomes.^3,19^

### Statistical Analysis

We explored the EPDS trajectories of women across 6 repeated assessments from 21 months to 11 years after childbirth using linear growth modeling and controlling for maternal education (Stata command: xtmixed, using mixed-effects maximum likelihood regression and unstructured covariance matrix; Stata, version 13 [StataCorp LLC]). We compared both the intercepts (overall mean EPDS scores across the repeated measures) and the slopes (the extent to which the scores increased or decreased over time, achieved by exploring the interaction between the PND and time variables). We present EPDS means and SDs at 21 months, 33 months, and 11 years.

We performed logistic and ordered logistic regressions, controlling for maternal education, to investigate the association between PND and the 3 child outcomes^18,19,20^ (child behavioral problems at 3.5 years of age, GCSE mathematics grades at 16 years of age, and offspring depression at 18 years of age). We examined different levels of severity and persistence to elucidate the contribution of these factors and to explore a potential dose-response relationship.

## Results

For the 9848 mothers in the sample, the mean (SD) age at delivery was 28.5 (4.7) years. Of the 8287 children, 4227 (51%) were boys and 4060 (49%) were girls.

### Long-term Course of Depression

Table 1 presents means and SDs of EPDS scores for the 3 levels of PND severity (moderate, marked, and severe) at 21 months, 33 months, and 11 years for mothers with PND that did or did not persist to 8 months.

**Table 1.** Mean EPDS Scores for Participants With Depression in the Postnatal Year

Level of PND Severity	2 mo After Childbirth (n = 9848)		8 mo After Childbirth (n = 9848)		21 mo After Childbirth (n = 8679)		33 mo After Childbirth (n = 8103)		11 y After Childbirth (n = 6182)	
	Mean (SD) Score	Participants, No. (%)	Mean (SD) Score	Participants, No. (%)	Mean (SD) Score	Participants, No. (%)	Mean (SD) Score	Participants, No. (%)	Mean (SD) Score	Participants, No. (%)
Below threshold^a^	4.71 (3.39)	8878 (90.2)	4.14 (3.27)	8878 (90.2)	4.78 (4.03)	7871 (90.7)	5.36 (4.37)	7409 (91.4)	5.03 (4.80)	5648 (91.4)
Moderate but not persistent^b^	13.42 (0.49)	300 (3.1)	7.64 (3.25)	300 (3.1)	8.17 (4.56)	260 (3.0)	9.63 (4.72)	229 (2.8)	9.09 (5.53)	163 (2.6)
Marked but not persistent^c^	15.44 (0.50)	158 (1.6)	8.97 (3.42)	158 (1.6)	10.75 (5.70)	130 (1.5)	10.48 (4.93)	112 (1.4)	9.08 (6.03)	87 (1.4)
Severe but not persistent^d^	19.06 (2.30)	225 (2.3)	10.26 (4.25)	225 (2.3)	11.07 (5.60)	184 (2.1)	10.87 (5.77)	143 (1.8)	9.93 (5.84)	125 (2.0)
Moderate persistent^e^	13.50 (0.50)	129 (1.3)	15.39 (2.96)	129 (1.3)	12.52 (5.00)	108 (1.2)	13.55 (5.00)	109 (1.4)	12.45 (5.64)	78 (1.3)
Marked persistent^f^	15.45 (0.50)	75 (0.8)	17.80 (3.14)	75 (0.8)	15.05 (5.09)	61 (0.7)	13.04 (5.34)	48 (0.6)	11.52 (5.32)	44 (0.7)
Severe persistent^g^	20.66 (3.05)	83 (0.8)	19.95 (2.67)	83 (0.8)	16.29 (4.98)	65 (0.8)	15.30 (6.19)	53 (0.7)	14.49 (6.13)	37 (0.6)

Abbreviations: EPDS, Edinburgh Postnatal Depression Scale; PND, postnatal depression.

^a^ EPDS score of less than 13 points in the postnatal year.

^b^ EPDS score of 13 to 14 points at 2 months and less than 13 points at 8 months.

^c^ EPDS score of 15 to 16 points at 2 months and less than 15 points at 8 months.

^d^ EPDS score of 17 or more points at 2 months and less than 17 points at 8 months.

^e^ EPDS score of 13 to 14 points at 2 months and 13 or more points at 8 months.

^f^ EPDS score of 15 to 16 points at 2 months and 15 or more points at 8 months.

^g^ EPDS score of 17 or more points at 2 and 8 months.

Mean scores remained relatively stable from 21 months to 11 years for women with persistent PND. The mean EPDS score of women with persistent moderate PND remained high at subsequent times: 12.52 (5.00) points at 21 months and 12.45 (5.64) points at 11 years. Similarly, the mean EPDS score for women with persistent severe PND remained high with little improvement even up to 11 years after childbirth: 16.29 (4.98) points at 21 months and 14.49 (6.13) points at 11 years.

Table 2 shows the analysis of the linear growth modeling indicating the mean EPDS scores across 6 time points (measured approximately yearly from 21 months to 11 years after childbirth) and the influence of PND severity and persistence on these trajectories. For the sample as a whole, the models indicate that, after the postnatal year, there was little change in the mean EPDS scores over time; mean EPDS scores rose by 0.004 points (95% CI, −0.02 to 0.03) at each repeated assessment. Compared with women who were not above the threshold in the postnatal year (EPDS score <13 points), all other groups had consistently higher EPDS scores up to 11 years after childbirth that progressed in a stepwise function. Compared with women in the reference group, women with an EPDS score of 13 to 14 points at 2 months only (moderate but not persistent PND) had higher EPDS scores of, on average, 3.46 points (95% CI, 2.86-4.05); women whose EPDS scores were 17 or more points at 2 months only (severe but not persistent PND) had higher EPDS scores of, on average, 5.84 points (95% CI, 5.13-6.55); women whose EPDS scores were 13 to 14 points at both 2 and 8 months (moderate and persistent PND) had higher EPDS scores of, on average, 6.91 points (95% CI, 5.98-7.83); and women whose EPDS scores were 17 or more points at both 2 and 8 months (severe and persistent PND) had higher EPDS scores of, on average, 9.90 points (95% CI, 8.73-11.08). There was no evidence that the change in scores (the slope) differed between women with depression that did not persist and women with depression that did persist, suggesting that EPDS scores did not improve over time and were consistently higher compared with women with no depression (Table 2).

**Table 2.** Mixed-Effects Linear Regression at Different Levels of Postnatal Depression

Level of PND Severity	Difference in Intercept of EPDS Scores in Postnatal Year, Coefficient (95% CI)	*P* Value	Increase in EPDS Scores at Each Assessment, Slope (95% CI)	*P* Value
Below threshold^a^	1 [Reference]	NA	1 [Reference]	NA
Moderate but not persistent^b^	3.46 (2.86 to 4.05)	<.001	0.11 (−0.01 to 0.23)	.06
Marked but not persistent^c^	4.77 (3.93 to 5.62)	<.001	−0.04 (−0.81 to 0.13)	.66
Severe but not persistent^d^	5.84 (5.13 to 6.55)	<.001	−0.10 (−0.24 to 0.045)	.19
Moderate persistent^e^	6.91 (5.98 to 7.83)	<.001	0.10 (−0.08 to 0.28)	.29
Marked persistent^f^	8.65 (7.48 to 9.83)	<.001	−0.19 (−0.42 to 0.50)	.12
Severe persistent^g^	9.90 (8.73 to 11.08)	<.001	0.10 (−0.14 to 0.35)	.42

Abbreviations: EPDS, Edinburgh Postnatal Depression Scale; NA, not applicable; PND, postnatal depression.

^a^ EPDS score of less than 13 points in the postnatal year.

^b^ EPDS score of 13 to 14 points at 2 months and less than 13 points at 8 months.

^c^ EPDS score of 15 to 16 points at 2 months and less than 15 points at 8 months.

^d^ EPDS score of 17 or more points at 2 months and less than 17 points at 8 months.

^e^ EPDS score of 13 to 14 points at 2 months and 13 or more points at 8 months.

^f^ EPDS score of 15 to 16 points at 2 months and 15 or more points at 8 months.

^g^ EPDS score of 17 or more points at 2 and 8 months.

### Child Outcomes

Table 3 presents the odds ratios (ORs) of adverse child outcomes using the 3 thresholds of PND severity for women whose PND did or did not persist.

**Table 3.** Logistic and Ordinal Logistic Regressions Investigating the Association Between Postnatal Depression and Adverse Child Outcomes, Controlling for Maternal Education

Level of PND Severity	Behavioral Problems at 3.5 y (n = 7917)^a^		Low GCSE Mathematics Grades at 16 y (n = 4941)		Offspring Depression at 18 y (n = 3486)	
	OR (95% CI)	*P* Value	OR (95% CI)	*P* Value	OR (95% CI)	*P* Value
Below threshold^b^	1 [Reference]	NA	1 [Reference]	NA	1 [Reference]	NA
Moderate but not persistent^c^	2.22 (1.74-2.83)	<.001	1.14 (0.77-1.68)	.51	1.11 (0.51-2.44)	.79
Marked but not persistent^d^	1.91 (1.36-2.68)	<.001	1.53 (0.89-2.63)	.13	2.34 (1.03-5.29)	.04
Severe but not persistent^e^	2.39 (1.78-3.22)	<.001	1.40 (0.89-2.22)	.15	1.72 (0.77-3.82)	.18
Moderate persistent^f^	3.04 (2.10-4.38)	<.001	1.65 (0.89-3.05)	.11	1.05 (0.32-3.42)	.94
Marked persistent^g^	2.84 (1.71-4.71)	<.001	1.32 (0.60-2.90)	.46	2.30 (0.67-7.90)	.19
Severe persistent^h^	4.84 (2.94-7.98)	<.001	2.65 (1.26-5.57)	.01	7.44 (2.89-19.11)	<.001

Abbreviations; EPDS, Edinburgh Postnatal Depression Scale; GCSE, General Certificate of Secondary Education; NA, not applicable; OR, odds ratio; PND, postnatal depression.

^a^ Using the Rutter revised total problems scale.

^b^ EPDS score of less than 13 points in the postnatal year.

^c^ EPDS score of 13 to 14 points at 2 months and less than 13 points at 8 months.

^d^ EPDS score of 15 to 16 points at 2 months and less than 15 points at 8 months.

^e^ EPDS score of 17 or more points at 2 months and less than 17 points at 8 months.

^f^ EPDS score of 13 to 14 points at 2 months and 13 or more points at 8 months.

^g^ EPDS score of 15 to 16 points at 2 months and 15 or more points at 8 months.

^h^ EPDS score of 17 or more points at 2 and 8 months.

#### Nonpersistent PND

For mothers with PND that was not persistent (ie, depression only at 2 months after childbirth), the risk of child behavioral disturbance at 3.5 years of age was somewhat raised; this risk was similar whatever PND severity threshold was applied. Thus, the OR for child behavioral disturbance for the maternal group with moderate PND was 2.22 (95% CI, 1.74-2.83), for the group with marked PND was 1.91 (95% CI, 1.36-2.68), and for the group with severe PND was 2.39 (95% CI, 1.78-3.22).

For the outcomes of GCSE mathematics grades at 16 years of age and offspring depression at 18 years of age, PND that was not persistent was not associated with increased risk. Furthermore, risk did not differ substantially between levels of severity with the exception of the group of mothers with marked PND, with offspring showing higher rates of depression at 18 years of age (OR, 2.34; 95% CI, 1.03-5.29).

#### Persistent PND

Children of women with persistent PND of moderate (OR, 3.04; 95% CI, 2.10-4.38) or marked severity (OR, 2.84; 95% CI, 1.71-4.71) were at higher risk of behavioral problems at 3.5 years of age compared with children of women with PND that was not persistent at any level of severity. The ORs for lower mathematics grades at 16 years of age and depression at 18 years of age were not substantially elevated in the context of either moderate or marked PND.

Compared with children of women with an EPDS score of less than 13 points in the postnatal year (reference group), children of women with persistent and severe depression were at the highest risk for all 3 adverse child outcomes (behavioral problems OR, 4.84 [95% CI, 2.94-7.98]; lower GCSE mathematics grades OR, 2.65 [95% CI, 1.26-5.57]; higher depression rate OR, 7.44 [95% CI, 2.89-19.11]).

## Discussion

We used a longitudinal prospective sample from a study with multiple assessments (ALSPAC) to examine the long-term course of PND and the association of PND at varying levels of severity and chronicity with child development. The adverse consequences on child development of severe and persistent PND is of particular note given the long-term follow-up of the children.

Using linear growth modeling, the data indicate that depression scores from 21 months to 11 years show relative stability. The data also indicate a step function, with higher mean depression scores for women whose PND persisted from 2 to 8 months after childbirth compared with women who scored below the threshold and those whose PND was not persistent. Further analysis indicated that, although the intercepts were higher for those with persistent PND, the slopes did not differ, suggesting that women with PND did not improve over time and women with persistent PND consistently remained at relatively higher EPDS scores. We found elevated risks for adverse outcomes for children of women who had persistent PND compared with women whose PND did not persist, and this association was especially pronounced in the group with persistent and severe PND. Postnatal depression that was not persistent either at moderate or marked severity level did not increase the risk in children for lower GCSE mathematics grades or offspring depression.

In practice, perinatal depression data are routinely collected in the United Kingdom, as recommended by the National Institute for Health and Clinical Excellence guidelines.^27^ Our results show that women who meet criteria for PND both early and late in the postpartum year are at an increased risk for prolonged depression. In addition, PND is most likely to raise the risk for adverse child development when PND is severe and persistent. Health care professionals should identify these women for further referral because early and effective treatment could reduce the continued exposure of the child. Owing to the frequent contact with health care professionals in the perinatal period, it is possible to identify women with persistent PND during this period. Identification of women with PND may be associated with increased treatment costs, but the overall cost to the public sector of perinatal mental health problems is 5 times more than the cost of improving services,^7^ further highlighting that early intervention and effective treatment of perinatal depression are a public health priority.

To date, the literature is mixed on whether treating maternal depression leads to positive child outcomes, particularly for depression in the early years of life. Treatments for PND have been relatively brief in duration and moderate in intensity; therefore, it is perhaps unsurprising that such interventions have not shown long-term benefits for either the mother or the child.^28,29,30,31^ A limited number of interventions targeting the mother-child relationship have shown some short-term benefits for outcomes, such as attachment and behavior.^32,33,34^ This finding highlights the complex issue of treatment recommendations, which may be further compounded by persistent and severe PND, which may in itself require more intensive treatment.

### Strengths and Limitations

A particular strength of our analysis is the large sample, which allowed us to categorize depressive symptoms in a stepwise function. However, the higher thresholds of marked and severe PND do not invariably reflect clinically severe depression, and thus further research using diagnostic instruments is needed. Other strengths of this study include the long-term follow-up and the inclusion of different outcomes at different ages, all of which confirm the increased risk for children of women with persistent and severe PND symptoms.

Our study was also subject to some limitations, including a relatively small number of women meeting criteria for persistent and severe PND. The ALSPAC has high attrition, especially during the later time points. The patterns of missing data suggest that children most disadvantaged and more likely to have mothers with depression are overrepresented in the group with missing data^20^; however, previous imputation analysis of the association between PND and mathematics grades and depression revealed that these associations did not attenuate following imputation.^19,20^ Women experiencing significant levels of depression may be more likely to opt out of the study. Therefore, our report on the proportion of women who experience severe and persistent PND may be an underestimate. Owing to the small sample size of women with persistent and severe PND, we decided a priori to control for maternal education only, as this is the demographic variable that has consistently been shown to influence the association between PND and child outcomes. Previous analyses showed no attenuation of the association between PND and child outcomes by including other potential confounders.^18,19,20^

The ALSPAC study did not collect information on whether women received psychological treatment, but the availability of such treatment at the time was severely limited. The available information indicates that less than 1% of the sample used antidepressants, which is too small a subsample for a subgroup analysis. Finally, women experiencing severe depressive symptoms may be more likely to report child behavioral problems.^35^ This observation is a potential limitation for 1 of our outcomes; however, we present objective and self-reported outcomes at 16 and 18 years of age indicating a similar pattern of risk when PND is persistent and severe.

## Conclusions

The analyses we conducted highlight that women with persistent depression in the postnatal year continue to experience elevated levels of depressive symptoms until at least 11 years after childbirth. Children of women with persistent PND, especially when it is severe, are at an increased risk for a number of adverse outcomes. Screening both early and late in the first postpartum year will enable the identification of women with persistent PND and thus the offer of appropriate treatment.^36^

## Notes

Supplement. **eAppendix.** Supplementary Material
