# Supplementary material for: Association of Persistent and Severe Postnatal Depression With Child Outcomes
Source: JAMA Psychiatry. 2018 Jan 31;75(3):247–53. doi: 10.1001/jamapsychiatry.2017.4363 (PMC5885957; doi:10.1001/jamapsychiatry.2017.4363)
Supplement: Supplement. — eAppendix. Supplementary Material [file jamapsychiatry-75-247-s001.pdf]

Supplementary Online Content

Netsi E, Pearson RM, Murray L, Cooper P, Craske MG, Stein A. Association of persistent and severe postnatal depression with child outcomes. *JAMA Psychiatry*. Published online January 31, 2018. 10.1001/jamapsychiatry.2017.4363

eAppendix. Supplementary Material

This supplementary material has been provided by the authors to give readers additional information about their work.

© 2018 American Medical Association. All rights reserved.

eAppendix.

Supplementary Material

Sample

ALSPAC is an ongoing population-based study investigating a wide range of environmental influences on the health and development of children. All pregnant women resident in the former Avon Health Authority in south-west England, having an estimated date of delivery between 1 April 1991 and 31 December 1992, were invited to take part. The children of 14,541 pregnancies were recruited in 1990-1992, increasing to 15,247 by the age of 18 by enrolling eligible children whose mother did not originally take part [1]. The representative nature of the original ALSPAC sample has been confirmed by comparison with the 1991 National Census data of mothers with infants under 1 year of age who were residents in the county of Avon. The study website contains details of all the data that are available through a fully searchable data dictionary (http://www.bris.ac.uk/alspac/researchers/data-access/data-dictionary/).

Offspring Depression

The CIS-R produces a diagnosis of depression from an algorithm based on ICD-10 criteria for a mild, moderate or severe episode of unipolar depression. To meet criteria, the participant must report at least one of two 'entry criteria' symptoms for at least two weeks: experiencing loss of pleasure and experiencing persistent low mood. At least two of seven possible associated symptoms are also required for a diagnosis (disturbed appetite, sleep problems, impaired motor responses, poor concentration, fatigue, poor self-esteem/negative cognitions, and suicidality). The CIS-R is widely used to detect common mental health disorders in the UK and has been used in the National Surveys of Psychiatric Morbidity.[2]

Maternal Education

Response categories were as follows: minimal education, compulsory secondary level (up to age 16), non-compulsory secondary (up to age 18), or University Level education.

eReferences.

1. Boyd A, Golding J, Macleod J, et al. Cohort Profile: The 'Children of the 90s'—the index offspring of the Avon Longitudinal Study of Parents and Children. *International Journal of Epidemiology*. 2013.
2. Fisher SD, Wisner KL, Clark CT, Sit DK, Luther JF and Wisniewski S. Factors associated with onset timing, symptoms, and severity of depression identified in the postpartum period. *Journal of Affective Disorders*. 2016; 203: 111-20.

© 2018 American Medical Association. All rights reserved.
